# Apoptosis in Cancer Cells Is Induced by Alternative Splicing of hnRNPA2/B1 Through Splicing of Bcl-x, a Mechanism that Can Be Stimulated by an Extract of the South African Medicinal Plant, *Cotyledon orbiculata*

**DOI:** 10.3389/fonc.2020.547392

**Published:** 2020-10-08

**Authors:** Tshepiso Jan Makhafola, Mzwandile Mbele, Kiren Yacqub-Usman, Amy Hendren, Daisy Belle Haigh, Zoe Blackley, Mervin Meyer, Nigel Patrick Mongan, David Owen Bates, Zodwa Dlamini

**Affiliations:** ^1^SA-Medical Research Council (MRC)/UP Precision Prevention & Novel Drug Targets for HIV-Associated Cancers Extramural Unit, Faculty of Health Sciences, Pan African Cancer Research Institute (PACRI), University of Pretoria, Pretoria, South Africa; ^2^Division of Cancer and Stem Cells, Centre for Cancer Sciences, Biodiscovery Institute, University of Nottingham, Nottingham, United Kingdom; ^3^School of Veterinary Medicine and Science, University of Nottingham, Nottingham, United Kingdom; ^4^Biolabels Unit, Department of Biotechnology, Department of Science and Technology (DST)/Mintek Nanotechnology Innovation Centre (NIC), University of the Western Cape, Bellville, South Africa

**Keywords:** colorectal cancer, esophageal cancer, *Cotyledon orbiculata*, hnRNPA2B1, Bcl-x, caspase-3, alternative splicing

## Abstract

Alternative splicing is deregulated in cancer and alternatively spliced products can be linked to cancer hallmarks. Targeting alternative splicing could offer novel effective cancer treatments. We investigated the effects of the crude extract of a South African medicinal plant, *Cotyledon orbiculata*, on cell survival of colon (HCT116) and esophageal (OE33 and KYSE70) cancer cell lines. Using RNASeq, we discovered that the extract interfered with mRNA regulatory pathways. The extract caused hnRNPA2B1 to splice from the hnRNPB1 to the hnRNPA2 isoform, resulting in a switch in the BCL2L1 gene from Bcl-xL to Bcl-xS causing activation of caspase-3-cleavage and apoptosis. Similar splicing effects were induced by the known anti-cancer splicing modulator pladienolide B. Knockdown of hnRNPB1 using siRNA resulted in decreased cell viability and increased caspase-3-cleavage, and over-expression of hnRNPB1 prevented the effect of *C. orbiculata* extract on apoptosis and cell survival. The effect of the hnRNPA2/B1 splicing switch by the *C. orbiculata* extract increased hnRNPA2B1 binding to Bcl-xl/s, BCL2, MDM2, cMYC, CD44, CDK6, and cJUN mRNA. These findings suggest that apoptosis in HCT116, OE33, and KYSE cancer cells is controlled by switched splicing of hnRNPA2B1 and BCL2L1, providing evidence that hnRNPB1 regulates apoptosis. Inhibiting this splicing could have therapeutic potential for colon and esophageal cancers. Targeting hnRNPA2B1 splicing in colon cancer regulates splicing of BCL2L1 to induce apoptosis. This approach could be a useful therapeutic strategy to induce apoptosis and restrain cancer cell proliferation and tumor progression. Here, we found that the extract of *Cotyledon orbiculata*, a South African medicinal plant, had an anti-proliferative effect in cancer cells, mediated by apoptosis induced by alternative splicing of *hnRNPA2B1* and *BCL2L1*.

## Introduction

Colorectal cancer (CRC) and esophageal cancer (EC) are ranked the third and eighth most common cancers worldwide, respectively. Colorectal cancer and EC have a global mortality rate of 5.8 and 5.3%, respectively (1, 2). In South Africa, CRC is the fourth most common cancer, with a low survival rate of 8.1% (3, 4). Esophageal cancer is responsible for the second highest number of cancer-related mortalities, and has a 5 year relative survival rate of 4% in metastatic disease (5).

Despite advances in chemotherapy for the treatment of cancer, costs, side effects, and development of resistance to chemotherapy are major obstacles particularly in the global south. Most chemotherapeutic agents used to treat colon and esophageal cancers e.g., fluoropyrimidines, cisplatin, oxaliplatin, irinotecan, carboplatin, epirubicin, and docetaxel have been shown to induce resistance in cancer cells. Most of these treatments are mitotic inhibitors focusing on either preventing cell division by inhibiting tubule formation (e.g., docetaxel) or inducing mitotic arrest by interfering with DNA replication (platins). Resistance to chemotherapy has led to the search for alternative effective treatments (6, 7).

Most regulatory genes involved in apoptosis are expressed in different isoforms with distinct functional activity and changes in splice site patterns play an important role in cancer development (8). In cancer cells, the process of alternative splicing in these regulatory genes is deregulated, contributing to the deregulation of apoptosis, development of tumors, progression and maintenance of cancer, and eventual metastases. Both CRC and EC are thought to be linked to genetic and epigenetic abnormalities resulting from deregulated alternative splicing of apoptosis genes (9–11). Several alternative splicing factors are overexpressed and highly mutated in different cancer tissues (12). Additionally, changes in the splicing patterns of various alternative splicing events were identified in tumors demonstrating a prominent role in carcinogenesis (10), and a potential prognostic indication (13). Developing new pharmaceuticals that target alternative splicing may create new therapeutic avenues for treating cancer. Various derivatives of natural products have shown potential for altering splicing to induce apoptosis. Examples include pladienolide B, a splicing inhibitor produced by *Streptomyces platensis* with potent cytotoxicity and antitumor activity both in cancer cell lines and mouse xenograft models (14, 15) and isoginkgetin, a biflavonoid isolated from *Ginkgo biloba* that inhibits tumor cell invasion and splicing both *in vivo* and *in vitro* (16). A derivative of pladienolide B is now in clinical trial for leukemia.

Medicinal plants are a source of numerous molecules with proven cytotoxicity and can trigger different signaling pathways in several types of cancers eventually inducing apoptosis (17, 18). Some of these molecules/compounds include Vinca alkaloids isolated from *Catharanthus roseus* and its derivatives vinblastine, vincristine, vinorelbine, vindesine (19, 20), podophyllin toxin isolated from *Podophyllum peltatum* and its derivatives teniposide and etoposide (21, 22), camptothecin isolated from *Camptotheca acuminate* and its derivatives topotecan, and irinotecan (23), and taxol isolated from *Taxus brevifolia* (23), and its derivatives paclitaxel, docetaxel, cabazitaxel (24, 25). All these drugs are widely used in first and second line cancer therapy, and have revolutionized the treatment of several solid tumors. Vinca alkaloids in particular represent the only therapeutic option for patients who show drug resistance or are not candidates for curative surgical interventions (26, 27). These are some examples highlighting the potential of plants as sources of anticancer drugs.

In this study, we evaluate the anticancer potential of *Cotyledon orbiculata* extract and use RNASeq to determine the primary mechanism of action. We identified that apoptosis and alternative splicing were key differentially regulated pathways as analyzed by gene ontology analysis, with RNA regulatory pathways being the primary pathway identified for all five types of alternative splicing. We therefore assessed the effect of the extract on global differential splicing (canonical and *de novo*) and identified significant changes in cancer related genes. Focusing on the effects on splicing of hnRNPA2B1 and BCL2L1, two genes highly altered, we identified a potential key regulatory pathway of hnRNPB1 isoform in apoptosis.

## Methods

### Cell Culture

Human HCT116 colorectal carcinoma, OE33 esophageal adenocarcinoma of the lower esophagus (Barrett's metaplasia) and KYSE70 poorly differentiated invasive esophageal squamous cell carcinoma resected from middle intra-thoracic esophagus cell lines, were purchased from ATCC. Cells were maintained in RPMI-1640 medium containing 10% FBS and 1% Glutamine in a humidified chamber containing 5% CO_2_.

### Extract Preparation

*Cotyledon orbiculata* plants were purchased from Van den Berg Garden Village (Western Cape Province, Stellenbosch, South Africa). The leaves of the plants were removed, washed with water, cut into small pieces and dried in a ventilated oven at 40°C for 120 h. The dried leaves were crushed in a blender. Two and half (2.5) liters of boiling water were added to 100 g crushed leaf material and the mixture was stirred for 16 h at room temperature. This water extract was filtered through Whatman filter paper (0.45 μm pore size), frozen in liquid nitrogen and subsequently freeze-dried. The dried extract was kept in a desiccator until further use. Stock solutions of the extract was prepared in DMSO at 10 mg/ml, which was stored at −20°C.

### Cytotoxicity Assays

The effects of the crude extract of *C. orbiculata* on viability of HCT116 colorectal cancer cells, OE33 and KYSE77 esophageal cancer cells were determined using WST-1 assay. Briefly, a sub-confluent culture of HCT116, OE33, and KYSE70 were harvested using trypsin-EDTA and centrifuged at 200 × g for 5 min and re-suspended in growth medium to 5 × 10^3^ cells/ml. A total of 200 μl of the cell suspension was pipetted into each well of columns 2–11 of a 96 well culture plate. The same amount of the growth medium was added to wells of column 1 and 12 to maintain humidity and minimize the edge effect. The plates were incubated at 37°C in a 5% CO_2_ incubator overnight until the cells were in the exponential phase of growth. After incubation, cells were then treated with 6 concentrations of the extract (100–3.125 μg/ml). Each dilution of the test sample was tested in quadruplicate in each experiment and the experiments were repeated three times. The plates were again incubated for 48 h at 37°C in a 5% incubator. A negative control (untreated cells) and positive control (cells treated with different concentrations of cisplatin) were included. After incubation, 20 μL of WST-1 reagent was added to each well and the plates were incubated for a further 1 h at 37°C. After incubation with WST-1, the plates were gently shaken and the amount of WST-1 reduction was measured immediately by detecting the absorbance using a microplate reader at a wavelength of 570 nm. The wells in column 1 and 12, containing medium and WST-1 but no cells were used to blank the microplate reader. The percentage of cell viability was calculated using the formula below:

$$\begin{array}{l} \%\text{cell viability }=\;(\text{mean absorbance of sample}/\\ \text{ mean absorbance of control})\times 100. \end{array}$$

The LC_50_ values (lethal concentration at which 50% of the cells are killed) were calculated as the concentration of the test sample that resulted in 50% reduction of absorbance compared to untreated cells.

### RNASeq Analysis

RNA was submitted to Deepseq for library preparation and next generation sequencing was completed on an Illumina NextSeq (Queen Mary University of London). Raw sequence reads in fastq format were processed for quality (phred scores <30 retained) and adapter trimmed using the Trimgalore wrapper for FastQC and Cutadapt (https://github.com/FelixKrueger/TrimGalore). The resultant quality-controlled reads were aligned to the GRCh38.83 genome build using STAR (28). Gene expression was quantified using FeatureCounts (29) and significantly differentially expressed genes identified using EdgeR and DESeq2 (30, 31). The rMATS tool (32) was used to determine the effect of the extract differential canonical and *de novo* splicing. Gene ontologies and gene set enrichments were determined using Webgestalt (33) and GSEA (34), respectively.

### Polymerase Chain Reaction

To assess the effects of *C. orbiculata* on splicing of hnRNPA2B1 and BCL2L1 in HCT116, OE33, and KYSE70 cancer cell lines, cells were treated with varying concentrations of the crude extract of *C. orbiculata* (LC_50_/2, LC_50_, LC_50_ × 2) for 48 h. Following treatment, total RNA was isolated from treated and untreated cells using TRI Reagent^®^ (Sigma). RNA quality and quantity were measured using a Spectrophotometer (NanoDrop2000, ThermoFisher Scientific) and samples were stored at ×80°C. Reverse transcription polymerase chain reaction (RT-PCR) was carried out using 1 μg RNA per sample to obtain a constant amount of cDNA using a Takara PrimeScript™ RT Reagent Kit (RR037A). Samples were denatured at 65°C for 10 min, followed by addition of 1 μl PrimeScript RT enzyme. Thereafter, samples were incubated at 25°C for 10 min, then 37°C for 60 min followed by enzyme inactivation at 85°C for 1 min. The cDNA was stored in −20°C for future use.

The cDNA samples were then subjected to PCR using GoTaq^®^ G2 Green Master Mix (Promega, M7822) and primers which amplify the different splice variants of hnRNPA2B1 and Bcl-xl/s (Table 1). GAPDH was used as a control. Primers were used at final concentration 0.4 μM with 1 μl cDNA (~50 ng). Samples were denatured at 96°C for 5 min, followed by multiple cycles (Table 1) of denaturation at 96°C for 30 s, annealing at primer specific temperature (Table 1) for 30 s, and extension at 72°C for 1 min. There was then a final extension step at 72°C for 10 min. Thereafter, the PCR products were subjected to electrophoresis. Samples were run on 2–3% agarose gels containing 50 ng/ml ethidium bromide for approximately 1 h at 90 V. Gels were visualized using a BioRad Gel Doc™ EZ System.

**Table 1:** Primers used to detect the splice variants of hnRNPA2B1 and Bcl-xl/s exposed to extract of *Cotyledon orbiculata*.

**Gene**	**Forward primer**	**Reverse primer**	**PCR conditions**
hnRNPA2B1	F1:ATGGAGAAAACTTTAGAAACTGT	GTTTCTTCACAGTCACATGGG	35 cycles, annealing 60°C
	F2:ATGGAGAGAGAGAAGGAACAGT		
Bcl-xl/s	CTGACATCCCAGCTCCACAT	AAGAGTGAGCCCAGCAGAAC	35 cycles, annealing 60°C
GAPDH	AATTCCATGGCACCGTCAAG	GGTCATGAGTCCTTCCACGA	28 cycles, annealing 60°C

### Immunocytochemistry for the Detection of Cleaved Caspase-3

To detect and quantify activation of caspase-3 in HCT116, OE33, and KYSE70 cancer cell lines following treatment with crude extract of *C. orbiculata*, cells were subjected to immunofluorescence staining. Cells were seeded at 5,000 cells per well in a black-walled, glass-bottomed 96 well plate (Corning) overnight and treated with varying concentrations of the plant extract based on the calculated LC_50_ value (LC_50_/2, LC_50_, LC_50_ × 2) for each cell line. Staurosporine and cisplatin were used as positive controls. The cells were washed with sterile 1 X PBS and fixed with 4% paraformaldehyde (PFA). After fixing, the cells were permeabilized with 0.2% TritonX, followed by blocking using 1% BSA for 1 h at room temperature. The cells were probed for cleaved caspase-3 (Cell Signaling Technology #9661). The cells were then washed with sterile 1 × PBS, then labeled with 4 μg/mL Alexa Fluor^®^ 488 goat anti-rabbit IgG secondary antibody (Product # A-11008, ThermoFisher Scientific, US) for 30 min in the dark. The cell nuclei and cytoskeleton were identified by counterstaining with DAPI and phalloidin. Samples were imaged using a Leica TCS SPE confocal microscope. The images were analyzed using Fiji (Image J 1.0) image analyzer software to calculate the amount of cleaved caspase-3 in cells, and estimate cell numbers. The red and blue channels were threshholded independently and the number of positive CC3 spots counted and divided by the number of DAPI positive nuclei. An analysis was done blind and automated.

Pladienolide B, a known mRNA splicing inhibitor and antitumor compound was used as a positive control for RT-PCR and immunocytochemistry experiments. Cells were treated with varying concentrations of Pladienolide B (0, 2.5, 5, and 10 nM) for 48 h.

### Knockdown and Over-Expression

The hnRNPB1 specific siRNA was designed using Sigma MISSION Custom siRNA. The sequence [hnRNPB1 anti sense oligo # 8811245892-000030, UUCCUCUCC AAAGGAACAG[dT][dT]]. Transfection was performed using Lipofectamine 2000 (Invitrogen) following the manufacturers instruction. Briefly, 2 μg of siRNA was mixed with the transfection reagent and added to HCT116 cells. Non-target (mock siRNA) and transfection reagent (alone) were included as controls. A time course of 24, 48, and 72 h was conducted to establish the time of optimal knockdown then 48 h taken forward for subsequent experiments. After 48 h, cells were collected for RNA extraction to be used for RT-PCR, fixed and stained for cleaved-caspase 3 or tested for effects on viability via cell8/WST-1 as described above. Over expression of GFP-tagged hnRNPB1 (Origene, Rockville MD) in HCT116 cells was achieved through plasmid transfection with FuGENE^®^ HD Transfection Reagent (Promega). HCT116 cells were seeded in a 6 well, tissue culture plate (150,000 cells/ml) and were allowed to attach. After attachment, the media was removed, cells were washed in PBS and trypsinised. Six (6) plates were used, 3 for RNA extraction at 24, 48 and 72 h after seeding and the remaining 3 for protein extraction at 24, 48, and 72 h after seeding. They were incubated at 37 degrees at 5% CO_2_ during this time. For each plate the cells in 2 wells were treated with medium, 2 were treated with vector DNA and 2 with the hnRNPB1 plasmid.

### Annexin V and Dead Cell Assay

To confirm whether *C. orbiculata* induces apoptosis in HCT116, OE33, and KYSE70 cancer cells, the FITC Annexin V/Dead cell Apoptosis kit (Invitrogen, ThermoFisher Scientific) was used. Cells (15 × 10^4^ cells/mL) were seeded in 6 well plates and treated with varying concentrations of the crude extract of *C. orbiculata* for 48 h. Cells were harvested, washed two times with ice cold PBS and adjusted at a density of 1 × 10^6^ cells/sample (500 μL). The cells were stained with FITC Annexin-V/PI staining kit according to the manufacturer's instructions. The cells were analyzed using Becton Dickinson and Company (FC500) flow cytometer. Results were analyzed using Kaluza analysis 1.5 software.

### RNA Immunoprecipitation

An RNA-binding protein immunoprecipitation (RIP) assay was conducted using the Magna RIP RNA-Binding Protein Immunoprecipitation Kit (Millipore) according to the manufacturer's protocol. Briefly, HCT116 colorectal cancer cells were treated with 70 μg/ml of *C. orbiculata* crude extract, collected and lysed by RIP lysis buffer. Untreated cells were used as a negative control. Subsequently, 100 μl cell extract was incubated with RIP buffer containing magnetic beads conjugated with human anti-hnRNPA2B1 antibody or negative control normal mouse IgG. Proteinase K was used to digest the protein and the immunoprecipitated RNA was purified. The isolated RNA was used for quantitative RT-qPCR analysis of the products of the *BCL2L1, BCL2, MDM2, cMYC, CD44, CDK6, cJUN*, and *TXNP* genes using LightCycler^®^ SYBR Green (Roche Life Science) qPCR kit following the manufacturer instructions. The selected mRNAs are involved in one way or another in apoptosis.

### Statistical Analysis

Data obtained from all experiments were analyzed using Graphpad Prism version 7.0 statistical software and the values are expressed as mean ± standard error of mean (SEM). Statistical significance was considered at *p* < 0.05 using the One-way ANOVA with *post-hoc* Dunnett's multiple comparisons test. The asterisk (^*^), (^**^), and (^***^) show *p* < 0.05, *p* < 0.01, and *p* < 0.001, respectively.

## Results

### *C. orbiculata* Reduces Viability of HCT116 Colon Cancer Cells, OE33 and KYSE70 Esophageal Cancer Cells

**Figure 1:**
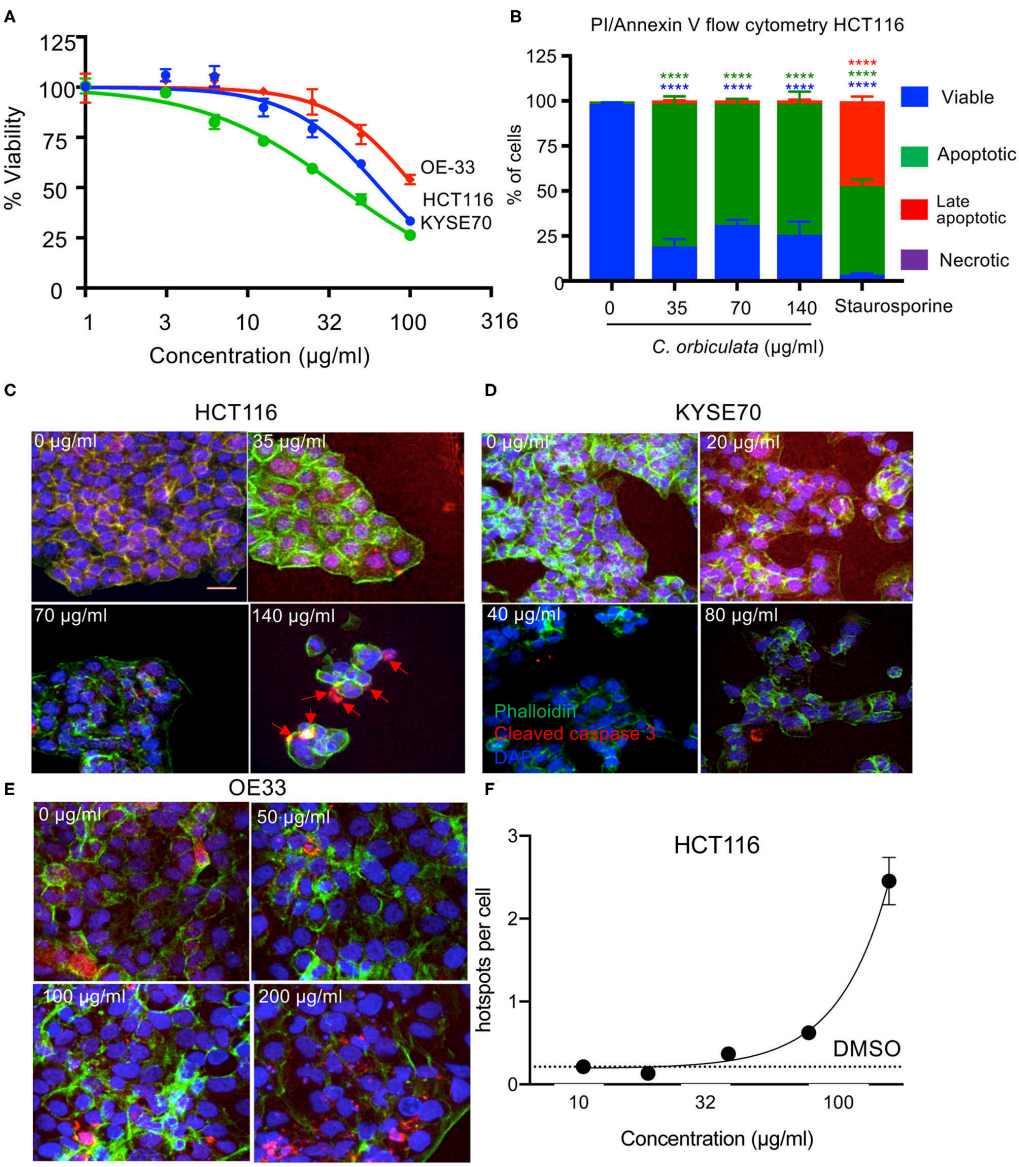
Decreased cell proliferation in HCT116, OE33, and KYSE70 cancer cells treated with varying concentrations of *C. orbiculata* for 48 h. The extract reduces viability of all three cancer cells in a dose dependent manner. **(A)** Cell viability was determined using the WST-1 cell viability assay (mean ± SEM, n = 3). **(B)** Percent of HCT116 cells analyzed by flow cytometry positive for Annexin V and propidium Iodide (PI). **(C)** Immunocytochemical analysis for apoptosis induction in HCT116, **(D)** KYSE70, and **(E)** OE33 cancer cells treated with varying concentrations of the crude extract of *C. orbiculata* (calculated from LC_50_ values) for cleaved caspase-3. Cells were fixed, permeabilised, and stained with DAPI (blue), phalloidin (green), and Caspase-3-cleavage antibody (red). There was an increase in cleaved caspase-3 per cell number in all three cell lines (e.g., red arrows); an indication of apoptosis induction. **(F)** Quantitative analysis of images shows a dose depended increase in cleaved caspase-3 in HCT116 cells. Results presented as caspase-3-cleavage/cell number, ± SEM (n = 3). Scale bar 25 μm. One-way ANOVA, Dunnett's multiple comparison test was used, ^*^P < 0.05, ^**^P < 0.01, ^***^P < 0.001, ^****^P < 0.0001. See also Supplementary Figure 1 for quantitation of KYSE70 and OE33.

The *in vitro* antiproliferative activity of the crude leaf extract of *C. orbuculata* against HCT116 colorectal carcinoma, OE33 esophageal adenocarcinoma and KYSE70 esophageal squamous carcinoma cell lines was evaluated using WST-1 cell viability assays at concentrations ranging from 100 to 3.1 μg/ml. The assay was carried out to determine the effective concentrations for use in subsequent experiments. The percentage cell viability results are shown in Figure 1. The WST-1 assay showed that the crude extract of *C. orbiculata* dose dependently decreased the viability of HCT116, OE33 and KYSE70 cancer cell lines with LC_50_ values of 64.9, >100, and 36.9 μg/ml, respectively (Figure 1A). The KYSE670 esophageal cancer cells were most susceptible to the extract, followed by HCT116 colorectal cancer cells and lastly OE33 esophageal cancer cells.

### *C. orbiculata* Induces Apoptosis in HCT116, OE33, and KYSE Cancer Cells

To determine the mechanism of cell death induced by *C. orbiculata* extract, we used Annexin-V/PI flow cytometry (Figure 1B), and found that programmed cell death (apoptosis, detected by an increase in the proportion of AnnexinV positive, PI negative cells), was induced after treatment with 35 μg/ml or above of the extract. Staurosporine at 10 nM was used as a positive control. To confirm that this was due to apoptosis, cleaved caspase-3, a key mediator of programmed cell death was stained for in HCT116, OE33, and KYSE70 cancer cells by immunocytofluorescence in cancer cells treated with varying concentrations of the plant extract (LC_50_ × 2, LC_50_, and LC_50_/2) (Figures 1C–E). Quantification showed an increase in the amount of cleaved caspase-3 in all three-cancer cell lines when comparing treated and untreated cells (Figure 1F, Supplementary Figure 1). Quantification of the images show a significant dose dependent increase in caspase-3-cleavage/cell number with up to a 12-fold increase in caspase-3-cleavage in HCT116 cancer cells treated with the plant extract when compared to the untreated cells (*p* < *0.0001* at 140 μg/ml) and up to a 2-fold increase in both OE33 and KYSE77 (Supplementary Figure 1).

### *C. orbiculata* Drives Alternative Splicing Pathways

**Figure 2:**
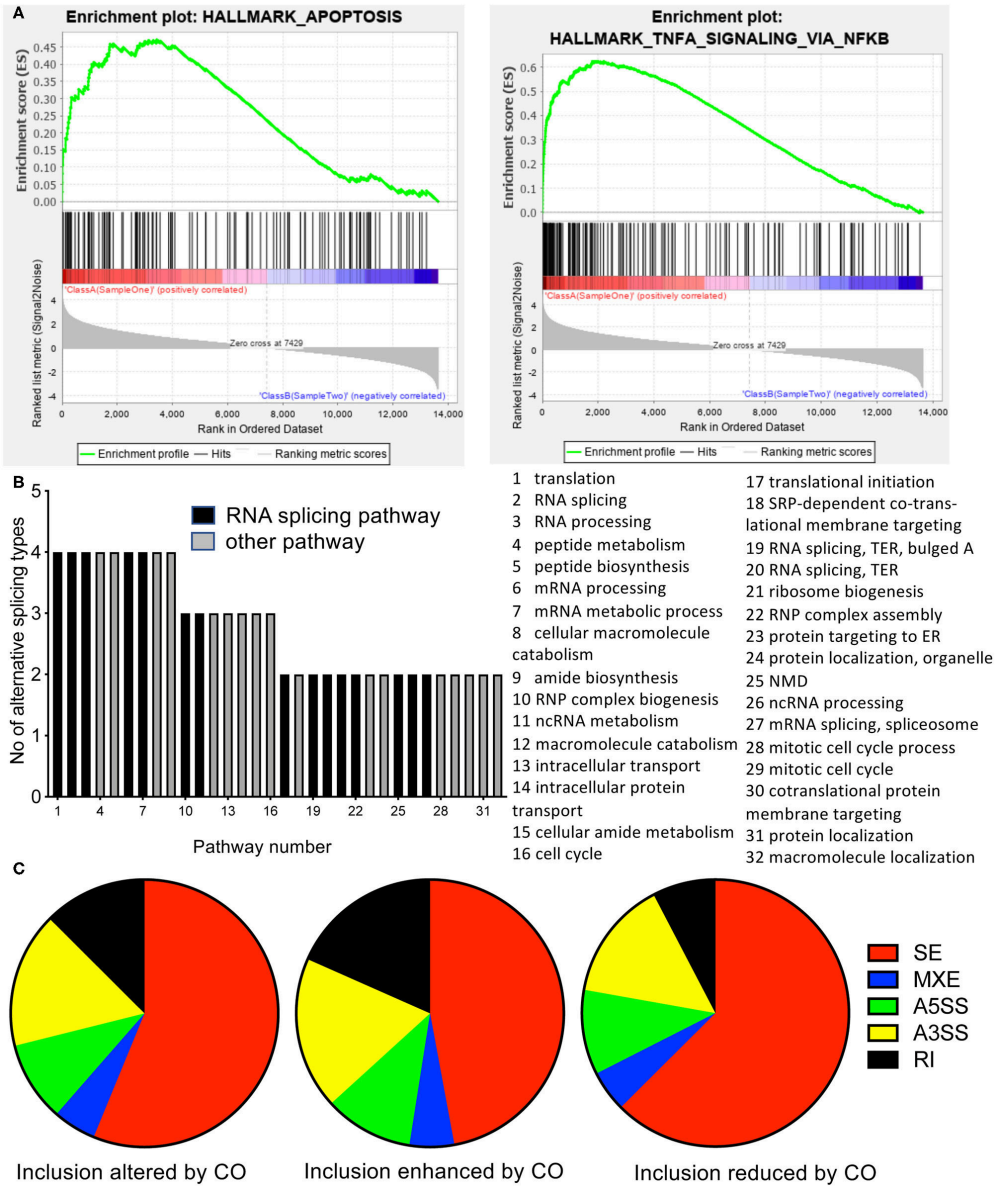
RNASeq analysis of cells treated with *C. orbiculata* extract. **(A)** Gene set enrichment analysis (GSEA) of RNA up or down regulated by treatment. Apoptotic and cell cycle genes were significantly altered. **(B)** Gene ontology analysis on altered splicing genes by gene set pathway analysis. Of the 15 pathways with the most significant change in percent spliced in (PSI) of the gene isoforms for each splice type the number of splice types are shown (i.e., 5 = the pathway was in the top 15 for all five splice types, 1 = the pathway was in the top 15 for only one splice type. **(C)** Distribution of gene splicing between the five types for isoforms altered either way, event included, or event excluded. SE, Skipped exon; MXE, mutually exclusive exons; A5SS, Alternative 5′ splice site; A3SS, alternative 3′ splice site; RI, retained intron.

To determine the mechanisms through which *C. orbiculata* drives apoptosis, we subjected HCT116 cells to 48 h treatment with 50 μg/ml of the extract, followed by RNA extraction, and RNASeq and differential gene expression (Supplementary Table 1) and differential isoform expression determined (data available on request). Gene ontology analysis of the RNASeq results based on expression showed that there was significant upregulation of various pathways, including movement of cell or subcellular components, inflammatory response, cell motility, localization of cell, and small molecule metabolic process at a significance of >10^−4^, but none of these pathways were linked to apoptosis or cell death, and the significance was not strong (gene ontology data available upon request). GSEA on Hallmarks of Cancer identified apoptosis and TNFα through NFκB as being significantly altered (Figure 2A). There was no obvious upregulation of genes involved in a coordinated manner to induce apoptosis. However, when we ran an analysis of splicing using rMATS, we identified by gene ontology analysis that RNA processing events were the most common pathways activated by *C. orbiculata* (Figure 2B, Supplementary Table 2). We examined the top 25 pathways ranked by False Discovery Rate (FDR) for each of the five types of splicing event—retained introns (RI), alternative 5′ and 3′ splice sites (A5SS and A3SS), mutually exclusive exons (MXE) and skipped exons (SE). Thirty-two (32) different pathways fell into this category. Of the 9 pathways that were significantly altered for four types of splicing event–5 were pathways involved in RNA regulation (Figure 2B). There were significant changes in splicing seen in all 5 categories of splicing. Of the 10,459 alternatively spliced events, 56% were skipped exons (Figure 2C). There were substantially fewer exons where exon inclusion was enhanced by *C. orbiculata* (45% of altered splicing events, Figure 2C), than exons inclusion was decreased by *C. orbiculata* (55%, Figure 2C), suggesting that *C. orbiculata* preferentially causes exon skipping. Retained introns were also significantly enhanced by *C. orbiculata* (from 7.6 to 18.4%).

### *C. orbiculata* Modulates Splicing of hnRNPA2B1 and BCL2L1 in HCT116, OE33, and KYSE Cancer Cells

**Figure 3:**
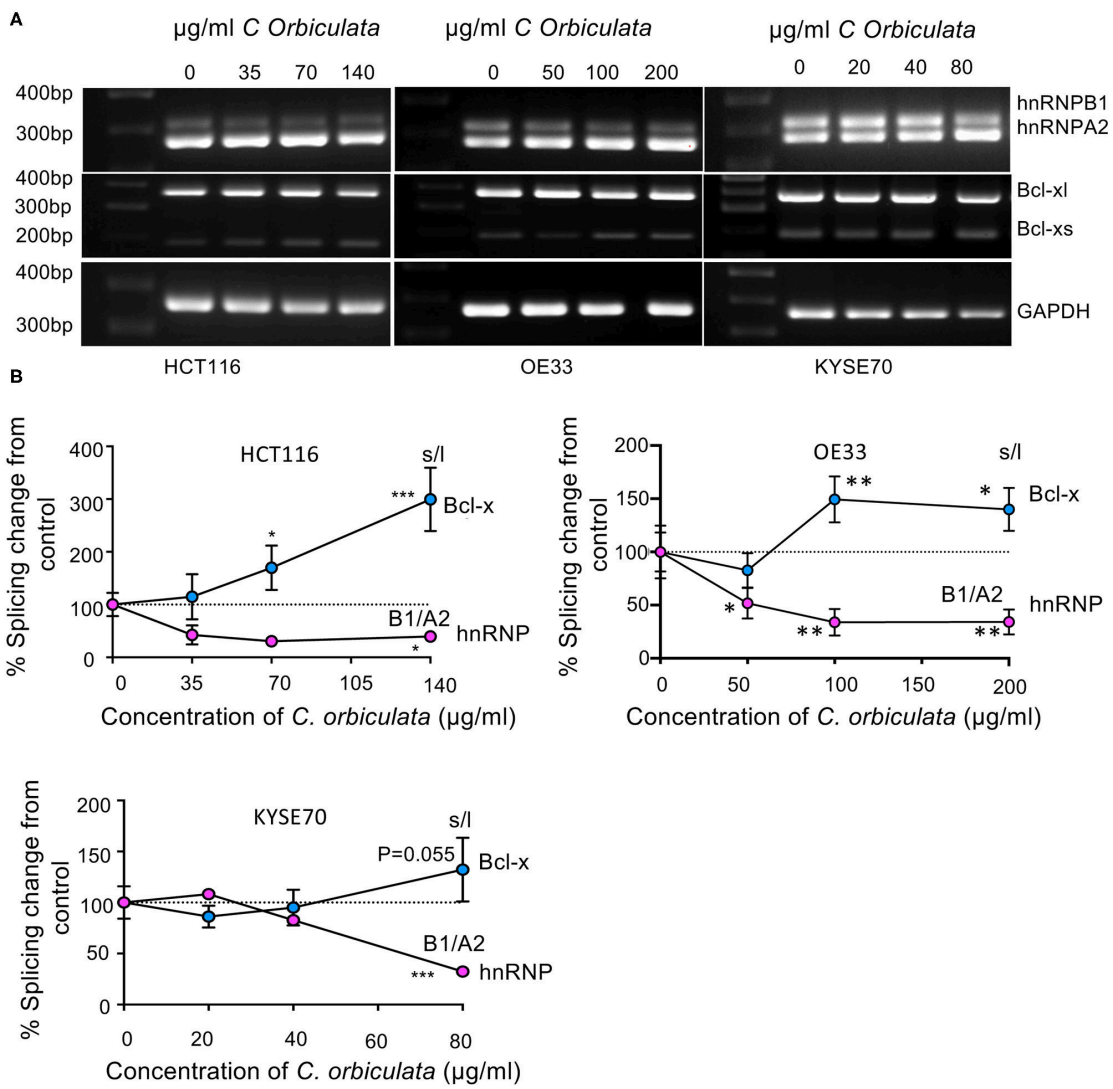
Effects of *C. orbiculata* on splicing of hnRNPA2B1 and BCL2L1 in HCT116, OE33 and KYSE cancer cells. **(A)** RT-PCR images and **(B)** quantitative analysis of PCR results. Percentage changes in ratio of hnRNPA2B1 and Bxl-xl/s from control. GAPDH was used as a loading control. Quantification demonstrates a decrease in hnRNPB1, and an increase in Bcl-xs. *n* = 3, mean ± SEM (*n* = 3). ^*^P < 0.05, ^**^P < 0.01, ^***^P < 0.001, ^****^P < 0.0001.

Two of the highly regulated genes detected in the splicing environment that have been linked to apoptosis and RNA splicing, respectively, were, B Cell Lymphoma 2 like protein 1 (BCL2L1), and hnRNPA2/B1. BCL2L1 encodes for two splice variants of the protein Bcl-x, a large isoform (Bcl-xl) and short isoform (Bcl-xs) differentiated by alternative 5′ splice site selection in exon 2. Inclusion of the distal splice site (i.e., BCl-xl) was reduced from 97 ± 1.5% to 89 ± 0.5% inclusion (*p* = 0.0055). hnRNPA2/B1 codes for two isoforms, hnRNPA2 and hnRNPB1. hnRNPA2B1 exon 2 exclusion (resulting in B1 expression) was induced by treatment with *C. orbiculata*, inclusion of the exon fell from 22 ± 2.4 to 16 ± 1.6% (*p* = 0.00182). Neither gene was statistically significantly altered when analyzed by EdgeR, but were significantly altered by DESeq2 analysis (treated was 67% of control, *p* = 0.004 for BCL2L1 and treated was 54% higher than control for hnRNPA2B1, *p* = 4.3 × 10^−18^). Total transcript levels (in counts) were *BCL2L1* 8,128 ± 1,214 control, 3,692 ± 327 treated, *hnRNPA2B1* 16,252 ± 2,783 vs. 28,585 ± 1,494). To verify this effect we carried out PCR for these two genes on cells treated with *C. orbiculata*. As shown in Figure 3, treatment of HCT116, OE33, and KYSE70 cancer cells with varying concentrations of *C. orbiculata* extract results in modulation of splicing of both hnRNPA2B1 and BCL2L1 (Figure 3A). The extract of *C. orbiculata* increased alternative splicing of hnRNPA2B1 in a dose dependent manner resulting in reduced expression of hnRNPB1 in all 3-cell lines (Figure 3A). The splicing of BCL2L1 was also affected resulting in an increase in Bcl-xs (Figure 3A). In all cases, the hnRNPB1 splice variant was found to be downregulated in a concentration dependent manner whilst Bcl-xs was upregulated in a concentration dependent manner in cells treated with the crude extract of *C. orbiculata* (Figure 3B), consistent with the RNASeq data. This switch in splicing resulted in a decrease in the ratio of hnRNPB1 to hnRNPA2 and an increase in the ratio of Bcl-xs to Bcl-xl in all 3-cancer cell lines (Figure 3B). These results suggest that *C. orbiculata* could be acting as a splicing modulator to induce apoptosis through hnRNPA2/B1 and BCL2L1.

### Pladienolide B, Like *C. orbiculata*, Modulates Splicing of hnRNPA2B1 and BCL2L1 to Induce Apoptosis in HCT116 Cancer Cells

**Figure 4:**
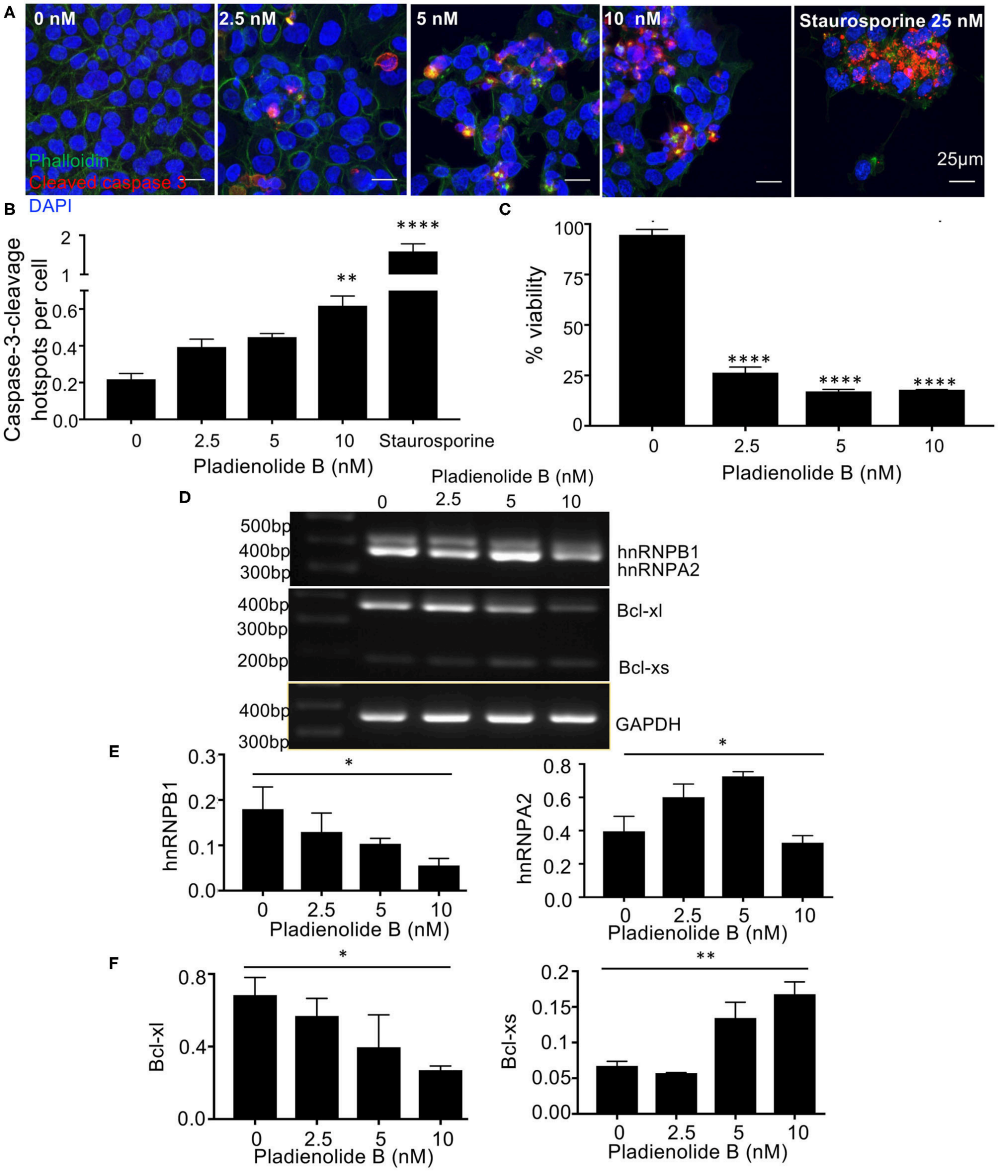
The splicing inhibitor and antitumor compound Pladienolide B switches splicing of hnRNPA2B1 and BCL2L1 to induce apoptosis in HCT116 colon cancer cells. **(A)** Cells were treated with varying concentrations of Pladienolide B for 48 h, fixed, permeabilized, and stained with DAPI (blue), phalloidin (green), and a Caspase-3-cleavage antibody (CCA) (red). **(B)** Increased caspase-3-cleavage and **(C)** Decreased cell viability in HCT-116 cells treated with Pladienolide B. The images were analyzed using Fiji to calculate the amount of cleaved caspase-3 in cells. mean ± SEM (*n* = 3). Scale bars 25 μm. **(D)** Effects of Pladienolide B on splicing of hnRNPA2/B1 and BCL2L1 in HCT116 colon cancer cells using semi quantitative PCR. **(E,F)** Quantitative analysis of PCR results. Quantification of splice variant expression demonstrates a decrease in hnRNB1, and an increase in Bcl-xs (*n* = 3, mean ± SEM), For statistical analysis, One-way ANOVA, Dunnett's multiple comparison test and *post-hoc* test for trend was used, ^*^P < 0.1, ^**^P < 0.01, ^***^P < 0.001, ^****^P < 0.0001.

To determine whether this specific switch in splicing is a common mechanism when splicing is altered, we used pladienolide B, a naturally occurring macrolide with both antitumor activity and mRNA splicing inhibition activities and measured the effect on splicing of these two genes. Pladienolide B dose dependently (0–10 nM) induced apoptosis in HCT116 colon cancer cells as evident by immunocytochemical analysis of caspase-3-cleavage (Figure 4A). The macrolide significantly increased caspase-3-cleavage/cell number up to 3-fold (*p* < 0.01, Figure 4B). Additionally, pladienolide B significantly decreased viability of HCT116 cancer cells at all tested concentrations ranging from 0 to 10 nM (*p* < 0.0001, Figure 4C). RT-PCR evaluation shows that pladienolide B switched splicing of hnRNPA2B1 and BCL2L1 (Figure 4D). Quantification of splice variant expression demonstrates a significant dose dependent decrease in hnRNB1 (*p* < 0.1), and a significant dose dependent increase in Bcl-xs (*p* < 0.1). Interestingly, pladienolide B, unlike *C. orbiculata* resulted in an increase in hnRNPA2 and a decrease in Bcl-xl shown in Figures 4E,F. These results suggest that the switch in splicing seen by *C. orbiculata* is consistent with it containing a naturally occurring splicing factor modulator, but that it could modulate a different pathway from Pladienolide B. hnRNPA2/B1 is a well-described splicing factor, having been shown to regulate multiple genes, including VEGF, Nav1.6, ANXA7 and the activity of kinases such as MEK. However, it is not clear whether the induction of the pro-apoptotic splice variant of BCL2L1 is independent of a splice form specific reduction in hnRNPB1 or a direct consequence of it. We therefore set out to determine whether hnRNPA2B1 could regulate BCL2L1 splicing.

### hnRNPB1 siRNA Modulates Splicing of BCL2L1, Inhibits Cell Viability and Induces Apoptosis in HCT116 Cancer Cells

**Figure 5:**
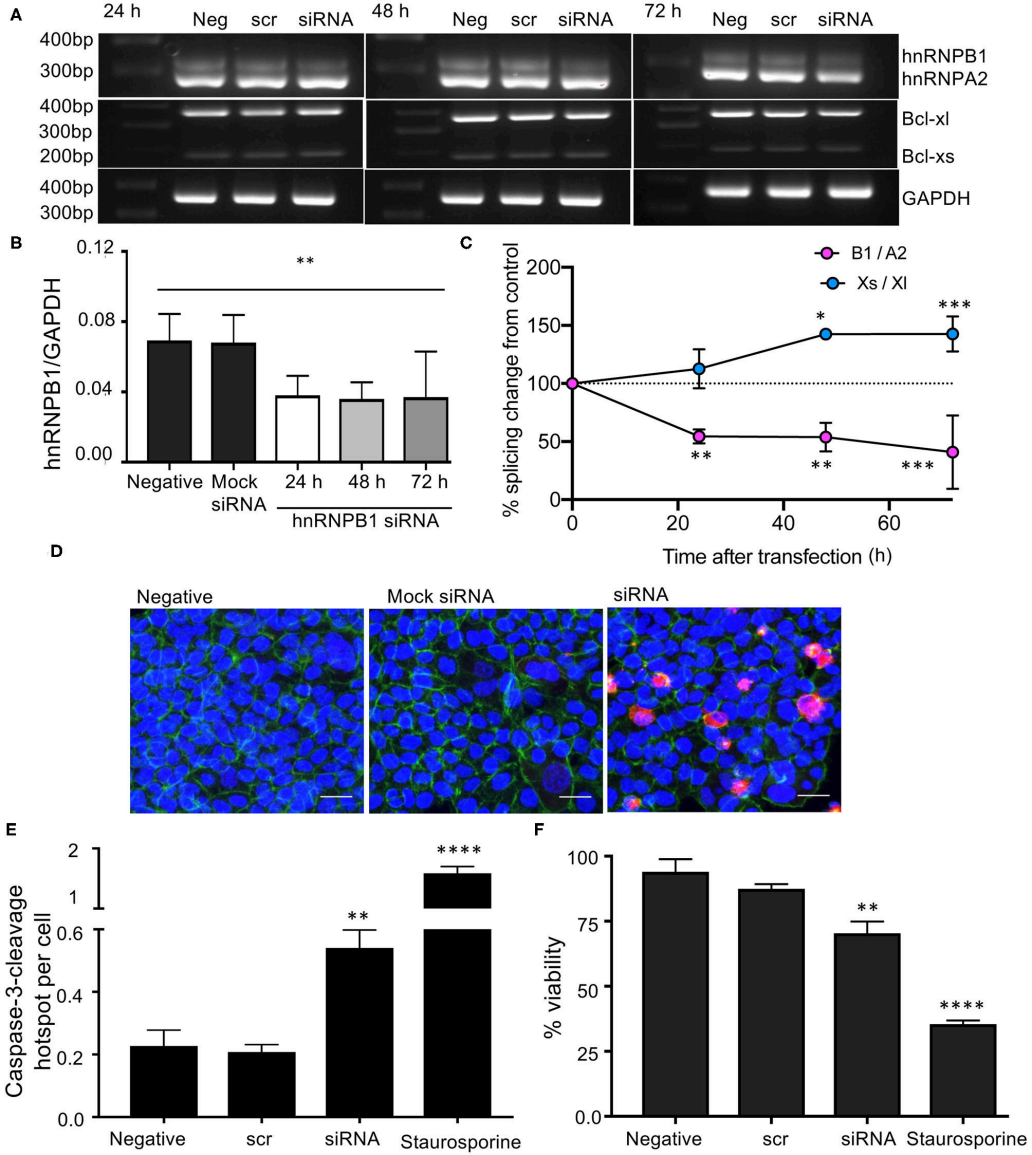
Knockdown of hnRNPB1 and its effects on splicing of BCL2L1 in HCT116 colon cancer cells. **(A)** Cells were transfected with 2 μg hnRNPB1 siRNA for 24, 48, and 72 h then RNA was extracted and subjected to RT-PCR. **(B,C)** Quantification of splice variant expression demonstrated a decrease in hnRNB1, and an increase in Bcl-xs (*n* = 3, mean ± SEM), **(D)** Increased caspase-3-cleavage and decreased cell viability in HCT-116 cells transfected with 2 μg hnRNPB1 siRNA for 48 h. Staurosporine was used as a positive control. DAPI (blue), phalloidin (green), and a Caspase-3-cleavage antibody (CCA) (red). Scale bar = 25 μm. **(E)** Quantification of the number of cells positive for cleaved caspase-3 mean ± SEM (*n* = 3). **(F)** Cell viability. For statistical analysis, One-way ANOVA with Dunnett's multiple comparison test and *post-hoc* test for trend was used, ^*^P < 0.1, ^**^P < 0.01, ^***^P < 0.001, ^****^P < 0.0001.

As *C. orbiculata* reduced the B1 isoform of hnRNPA2B1, we mimicked this by siRNA knockdown specifically of the hnRNPB1 isoform in HCT116 colon cancer cells. Cells were transfected with 2 μg of siRNA. The most effective silencing was at 48 h post transfection (Figures 5A,B). The knockdown of hnRNPB1 also caused a switch in splicing of BCL2L toward Bcl-xs (Figures 5A,C). Quantification of splice variant expression shows a decrease in hnRNB1 and an increase in Bcl-xs when comparing untransfected cells with cells transfected with 2 μg of hnRNPB1 siRNA (Figures 5B,C). This indicates that hnRNPB1 modulates splicing of BCL2L1. The transfection experiment also showed that induction of apoptosis in HCT116 colon cancer cells is replicated by isoform specific knockdown of hnRNPB1 using siRNA for 24, 48, and 72 h (Figure 5D). Silencing hnRNPB1 resulted in a significant increase in caspase-3-cleavage when comparing transfected cells to wild type/untransfected cells (*p* < 0.01, Figure 5E). There was up to a 3-fold increase in caspase-3-cleavage/cell number in transfected cells. Additionally, using WST-8/cell 8 cell viability assay, we found that hnRNPB1 siRNA significantly inhibited cell viability in transfected cells (*p* < 0.01, Figure 5F).

### *C. orbiculata* Mediated Cell Death Is hnRNPB1 Dependent

**Figure 6:**
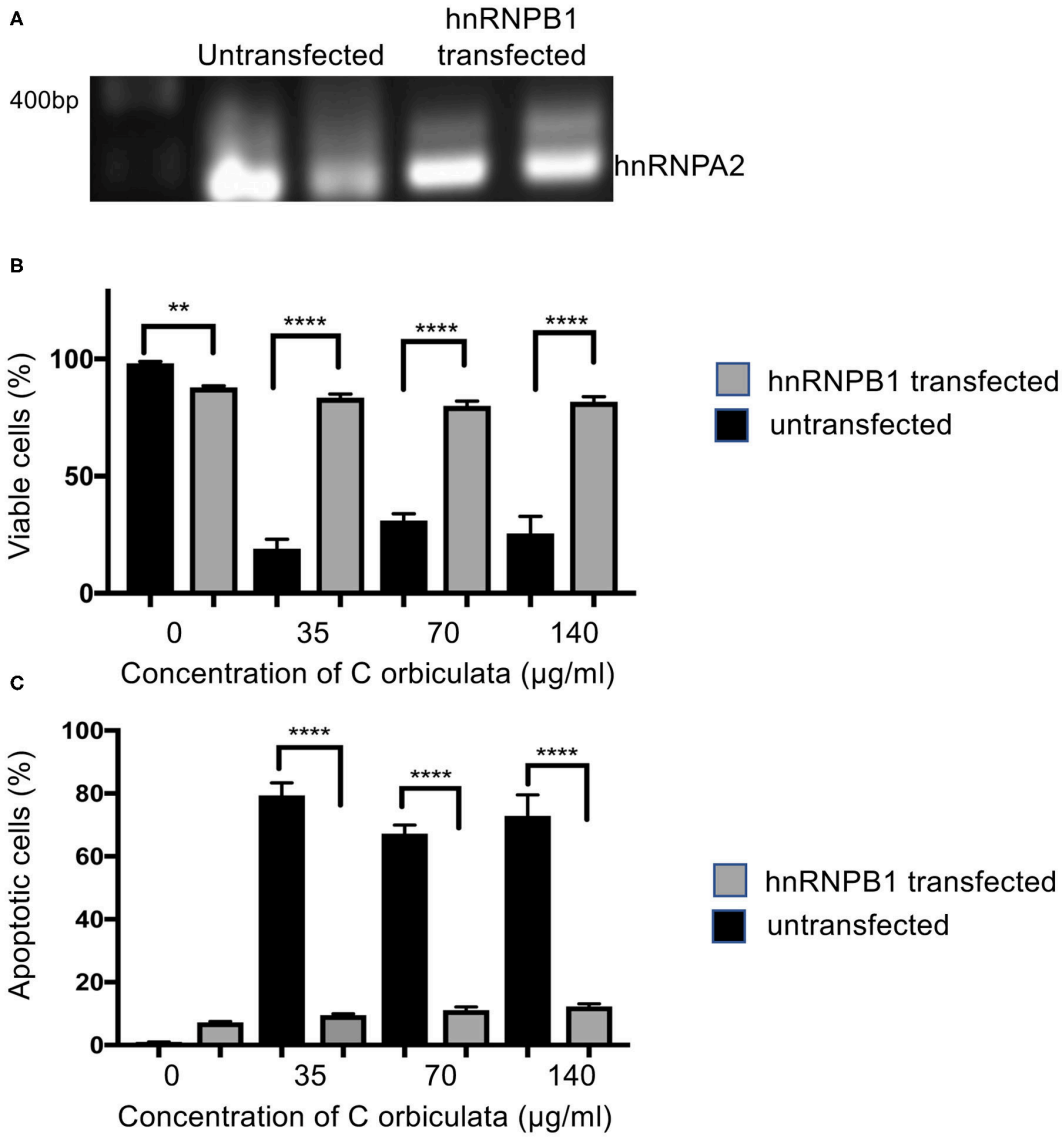
Overexpression of hnRNPB1 counteracts the effects of *C. orbiculata*. **(A)** Cells were transfected with 2 μg plasmid expressing hnRNPB1 cDNA and then RNA was extracted and subjected to RT-PCR. Cells were subjected to treatment with *C. orbiculata* for 48 h and then subjected to Annexin V/PI flow cytometry. **(B)** The proportion of viable cells was slightly lower in untreated cells transfected with hnRNPB1 plasmid. However, when treated with *C. orbiculata* extract there was a much reduced decrease in viability compared with untransfected cells. **(C)** The proportion of apoptotic cells was significantly reduced by transfection with hnRNPB1. One-way ANOVA with Dunnett's multiple comparison test and *post-hoc* test for trend was used, ^*^P < 0.05, ^**^P < 0.01, ^***^P < 0.001, ^****^P < 0.0001.

To confirm that the *C. orbiculata* extract was inducing apoptosis through switching splicing from hnRNPB1 we measured the effect of treatment after over-expression of hnRNPB1 isoform in HCT116 cells. Fourty eight (35) hours after transfection with hnRNPB1 plasmid, RNA was extracted, DNAse treated and subjected to RT-PCR. Figure 6A shows that cell transfected with the hnRNPB1 plasmid had increased hnRNPB1 expression as expected. When cells were treated with *C. Orbiculata* extract there was a small, significant reduction in the percent of viable cells as measured by PI-Annexin V flow cytometry, but this was much less than that induced in the wild type (Figure 6B). This protection from *C. orbiculata* extract was due to a decrease in apoptosis as measured by annexin V positivity in the transfected cells compared with the wild type (Figure 6C). These results clearly demonstrate that *C. orbiculata* extract induces apoptosis through reduction in the hnRNPB1 isoform.

### *C. orbiculata* Increases Association/Binding of hnRNPA2B1 to mRNAs Associated With Apoptosis

**Figure 7:**
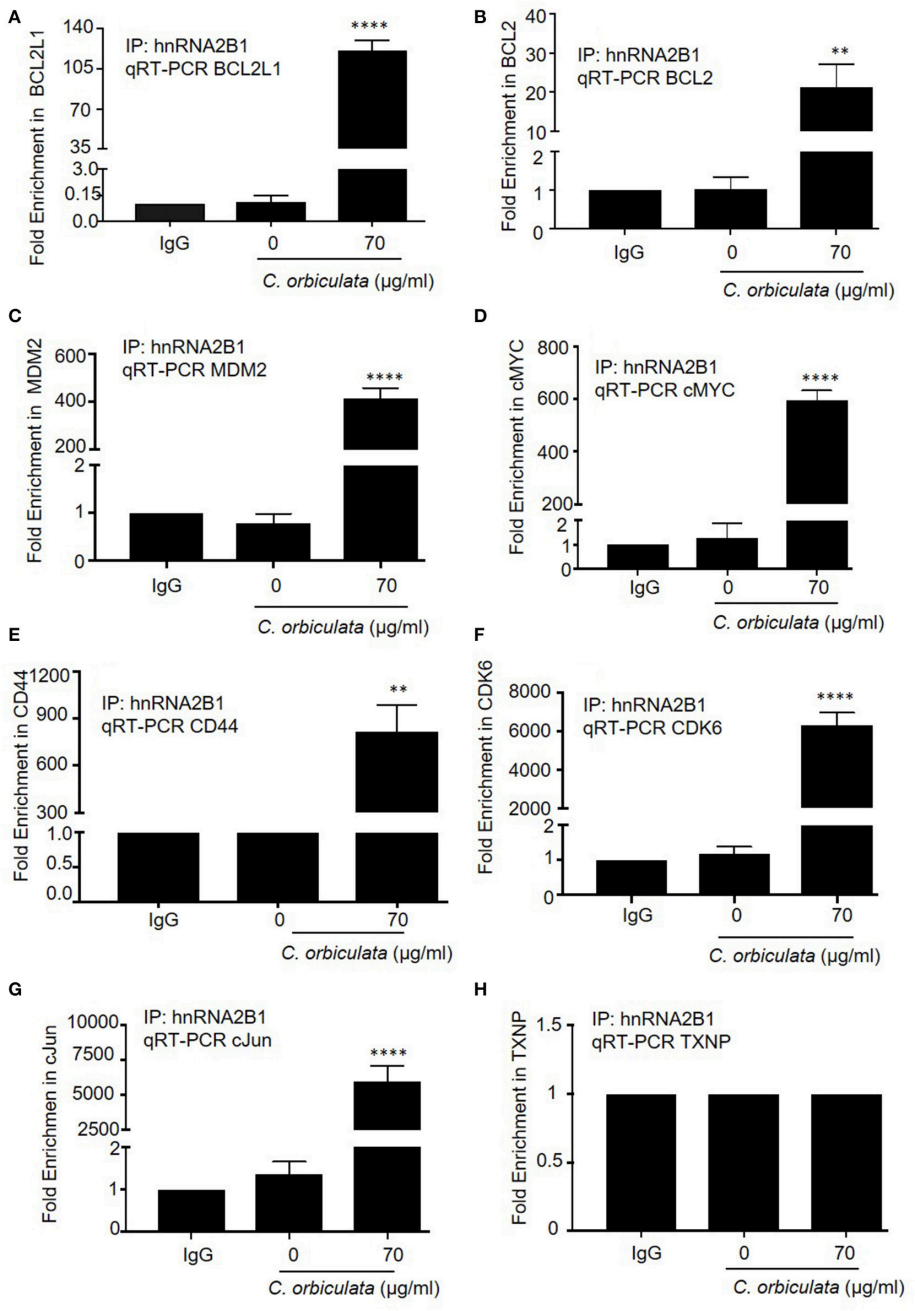
RNA-Binding Protein Immunoprecipitation RIP to detect interaction of hnRNPA2B1 with **(A)** BCL2L1, **(B)** BCL2, **(C)** MDM2, **(D)** cMYC, **(E)** CD44, **(F)** CDK6, **(G)** cJun, and **(H)** TXNP. Lysates prepared from HCT116 colon cancer cells treated with 70 μg/ml *C. orbiculata* were subjected to immunoprecipitation using 5 μg of either a normal mouse IgG or Anti-hnRNPA2B1 antibody. Successful immunoprecipitation of hnRNPA2B1-associated RNA was verified by qPCR using primers specific for the 8 mRNAs. Results are presented as fold increase relative to IgG. There is a clear significant increase in the binding of 7 **(A–G)** of the 8 mRNAs to hnRNPA2B1 in cells treated with the crude plant extract (*n* = 3, mean ± SEM). For statistical analysis, One-way ANOVA with Dunnett's multiple comparison test was used, ^*^P < 0.1, ^**^P < 0.01, ^***^P < 0.001, ^****^P < 0.0001.

Switching splicing of hnRNPA2B1 from the B1 to the A2 isoform should be able to alter the specific RNAs that the protein can interact with. We therefore determined the effect of *C. orbiculata* crude extracts on association of hnRNPA2B1 with selected mRNAs known to be either regulated, or involved in apoptosis (Table 2), by RNA- immunoprecipitation (RIP) assay. We evaluated the association of hnRNPA2B1 with BCL2L1, BCL2, MDM2, cMYC, CD44, CDK6, cJUN, and TXNP in HCT116 colon cancer cells treated with 70 μg/ml of the crude extract. Treatment of HCT116 colon cancer cells with 70 μg/ml of *C. orbiculata* extract resulted in an increase in the co-precipitation of hnRNPA2B1 with BCL2L1 (Figure 7A), BCL2 (Figure 7B), MDM2 (Figure 7C), cMYC (Figure 7D), CD44 (Figure 7E), CDK6 (Figure 7F), and cJUN (Figure 7G) mRNA. The highest fold enrichment was observed for CDK6 (6,333-fold), followed by cJun (5,947-fold), CD44 (817-fold), cMYC (595-fold), MDM2 (416-fold), and BCL2L1 (120-fold) in HCT116 cells treated with *C. orbiculata*. There was no change in the binding of TXNP (Figure 7H). These results clearly show that a reduction in the hnRNPB1 isoform by *C. orbiculata* extract changes the interaction with multiple RNAs of genes associated with apoptosis, indicating that hnRNPA2B1 is a key regulator of apoptosis through its RNA binding activity and effect on alternative splicing.

**Table 2:** Primers used to detect mRNAs hnRNPA2B1-RIP lysates using qPCR analysis following exposure to extract of *Cotyledon orbiculata*.

**mRNA/gene**	**Primer sequences**
BCL2L1	FP 5′-CTGACATCCCAGCTCCACAT 3′ RP 5′ AAGAGTGAGCCCAGCAGAAC 3′
BCL2	FP 5′ CAGGAGAATGGATAAGGCAAA 3′ RP 5′ CCAGCCAGATTTAGGTTCAAA 3′
MDM2	FP 5′ GACTCCAAGCGCGAAAAC 3′ RP 5′ CAGACATGTTGGTATTGCACATT 3′
cMYC	FP 5′ AAGGGTGTTGGGTCTCCTG 3′ RP 5′ GTCTGTGTGGCCGCTGTT 3′
CD44	FP 5′ CAACAACACAAATGGCTGGT 3′ RP 5′ CTGAGGTGTCTGTCTCTTTCATCT 3′
CDK6	FP 5′ TGATCAACTAGGAAAAATCTTGGAC3′ RP 5′ GGCAACATCTCTAGGCCAGT 3′
cJun	FP 5′ TTCTATGACGATGCCCTCAACGC 3′ RP 5′ GCTCTGTTTCAGGATCTTGGGGTTAC 3′
TXNP	FP 5′ ATTCCAACATGGTATTCTTTGAAGT 3′ RP 5′ CCCACTTTTGTCCCTTCTTAAA 3′

## Discussion

Evidence suggests that alternative splicing of mRNA encoding proteins involved in apoptosis play a major role in the development and progression of cancer. BCL2L1 is a member of the BCL-2 gene family, which regulates apoptotic cell death in various cells. BCL2L1 is alternatively spliced to produce two isoforms, the longer anti-apoptotic Bcl-xl protein and the shorter pro-apoptotic Bcl-xs isoform with opposite effects on apoptosis (36). Bcl-xl is the predominant form in cancer cells, and its overexpression confers resistance to chemotherapeutic agents (37). The regulation of BCL2L1 alternative splicing is of critical importance to the apoptotic process and is highly relevant to cancer. In a number of cancers and cancer cell lines, the expression of the anti-apoptotic protein Bcl-xl is increased, and the ratio of the splice variants is frequently shifted to favor production of Bcl-xl. Bcl-x regulates the permeability of the outer membrane of mitochondria. The Bcl-xs splice isoform is proapoptotic whereas the Bcl-xl isoform is antiapoptotic as it prevents the release of mitochondrial components that would lead to apoptosis (38).

Bcl-xl exerts anti-apoptotic activity by forming heterodimers with both BAX and BAK. The pro-apoptotic function of Bcl-xs is derived from its capacity to disrupt the complex between BAK and the voltage-dependent anion channel (VDAC) protein by interacting with VDAC, thus freeing BAK for activation (39, 40). The overexpression of Bcl-xl has been shown to protect cells from TNF-mediated apoptosis and is involved in the inflammatory response by inhibiting the activation of NF-κB (41). Bcl-xl has also been shown to have an apoptosis-independent function in metastasis in pancreatic neuroendocrine tumor and breast cancer cell lines via nuclear promotion of epithelial-mesenchymal transition, migration, invasion, and stemness and in chemoresistance via RAS interaction and influence on EMT and regulation of cancer-initiating cell (CICs) (42, 43).

The control of splicing of BCL2L1 has not been clearly shown. Here we identify through knockdown of hnRNP that this results in a switch in splicing from the anti- (Bcl-xl) to the pro – (Bcl-xs) apoptotic isoform, and induction of apoptosis showing that the hnRNPB1 isoform is anti-apoptotic, suggesting that the A2 isoform is pro-apoptotic. This is consistent with the known activities of the hnRNPA2/B1 gene, but for the first time identifies the mechanism through which this acts, binding directly to BCL2L1 RNA and inducing alternate splicing.

hnRNPA2B1 is implicated to play a direct role in cancer development, progression, gene expression, and signal transduction and has been proposed to account for the oncogenic effects of several types of cancers (44). hnRNPA2B1 belongs to a family of heterogeneous nuclear ribonucleoproteins (hnRNPs) which are RNA-binding proteins involved in RNA processing, pre-mRNA splicing, mRNA export, localization, translation, stability, DNA repair, telomere biogenesis, cell signaling, and the regulation of gene expression. The levels of hnRNPA2B1 expression are associated with poor prognosis and disease progression (45). hnRNPA2B1 is alternatively spliced by cassette exon exclusion of hnRNPB1, containing exon 2, constitutes 2–5% of hnRNPA2B1 mRNA with the remainder alternatively spliced into hnRNPA2 lacking exon 2. Some studies have demonstrated that hnRNPA2B1 increases the tumorigenic potential of cells by regulating signal transducer and activator of transcription 3 (STAT3) and extracellular-signal-regulated kinase 1/2 (ERK1/2) signaling pathway (44). The overexpression of hnRNPA2 in immortal cells results in malignant transformation, suggesting that hnRNPA2 is a putative proto-oncogene. Some of the tumor suppressors and oncogenes regulated by HNRNPA2B1 include c-FLIP, BIN1, and WWOX, and the proto-oncogene RON (46).

hnRNPB1 has been proposed to be a tumor marker for human lung cancer (47, 48). hnRNPA2B1 is overexpressed in human gastric cancer, breast cancer cells, pancreatic cancer cells, lung cancer cells and several other cancers (35, 44). Interestingly, there are few studies on the involvement of hnRNPA2 or hnRNPB1 splicing differences in cancer. One report showed overexpression of hnRNPB1 correlated with the eventual development of lung cancer (49) and the mRNA is reported to be elevated in lung cancer tissue and it has been hypothesized to play a role in early carcinogenesis from a histological study of lung cancer specimens (50), but there is no clearly defined role of hnRNPB1 in cancers.

Inhibition of splicing has been shown to affect multiple hallmarks of cancer, including tumor cell proliferation and apoptosis. Thus, inhibitors of splicing have been intently looked for over the last 10 years and a few highly effective inhibitors have been identified. Natural product derivatives of *Streptomyces* bacteria such a pladienolide B or herboxidiene or isoginkgetin, a biflavonoid isolated from *Ginko biloba*, are inhibitors of the SF3B1 splicing factor that is required for constitutive splicing. Such compounds are generally toxic to most cells as they interfere with all splicing reactions, but tumor cells appear to be highly dependent on such constitutive splicing and so this chemotherapeutic approach has been taken into clinical trial with the SF3b targeting agent E7107 (51). Here we describe a novel mechanism of inhibiting splicing of key regulatory genes, in particular hnRNPA2B1 splicing, but also a number of others identified by RNASeq by an extract from the South African medicinal plant, *Cotyledon orbiculata*, or Pig's Ear. RNA Seq analysis of cells treated with this extract resulted in a high concentration of splicing factor, mRNA processing and mRNA transport mechanisms in the pathway analysis of alternative splicing variant. We identified that two key regulated RNA species were hnRNPA2/B1 and BCL2L1. While overall expression of these RNAs did not appear to be significantly altered in one analysis it was in another and expression of the hnRNPA2/B1 gene had increased by 75 ± 5%. Furthermore, there was a significant switch in isoform expression as revealed by RNASeq. It is possible that the significant heterogeneity of transcript/isoform expression observed may mask detection of significant changes in gene expression, given how transcript expression is compiled and assigned in gene level quantification. Future studies should therefore consider the complexity of transcript heterogeneity induced by *Cotyledon orbiculata* extract.

*Cotyledon orbiculata* is a small shrub with fleshy leaves belonging to the *Crassulaceae* family. It is widely distributed in Southern Africa and is used to treat a broad range of ailments. The leaves have been utilized to treat corns and warts (52, 53), the sap of the leaves is utilized as drops for toothache and earache; and is used as a hot poultice for boils and inflammation. The plant is also used to treat epilepsy (54).

hnRNPA2B1 has been proposed as a therapeutic target for anticancer therapy, but there are no previous reports of naturally occurring agents that can switch on targeting splicing to switch off B1 in colon cancer. The crude extract of *C. orbiculata* dose dependently reduced viability of colon and esophageal cancer cells. We found that the mode of cell death was apoptotic as evident by caspase-3-cleavage using immunocytochemistry. Caspase-3 is a major executioner caspase and its activation is a hallmark of apoptosis and can be used to quantify activators and inhibitors of the “death cascade” (55). To further confirm that *C. orbiculata* induces apoptosis in HCT116 colon cancer cells, we used Annexin-V-FITC and PI using flow cytometry to quantify the percentage of viable, apoptotic, late apoptotic and necrotic cells in HCT116 cells treated with varying concentrations of *C. orbiculata*. The extract resulted in an increase in apoptotic cells as evident by annexin-V-positive cells in the scatter plots and reduced viable cells when comparing treated cells to untreated cells at all tested concentrations (56).

We went on to evaluate the effects of *C. orbiculata* on splicing of hnRNPA2B1 and BCL2L1 in three-cell lines. The plant extract switched splicing of hnRNPA2B1, resulting in a decrease in the expression of hnRNPB1 mRNA and an increase in the expression of hnRNPA2. These changes in the splicing of hnRNPA2B1 resulted in an increase in the ratio of hnRNPA2 to hnRNPB1. We found that in addition to modulating the splicing of hnRNPA2B1, the splicing of BCL2L1 mRNA was also affected in cells treated with the extract of *C. orbiculata*. There was a clear increase in the expression of Bcl-xs isoform and almost no change in the expression of Bcl-xl in cells treated with varying concentrations of *C. orbiculata* when compared to untreated cells. These changes in splicing resulted in an increase in the ratio of Bcl-xs to Bcl-xl. The effects of *C. orbiculata* was similar to those of pladienolide B (14, 15) in that, just like *C. orbiculata*, pladienolide B switches splicing of hnRNPA2B1 and BCL2L1 to induce apoptosis in HCT116 colon cancer cells (Figure 4). However, pladienolide B also upregulated hnRNPA2, which *C. orbiculata* extract did not. While further work would need to be done to confirm this, including isolation of the active ingredient of *C. orbiculata*, and comparison of effects at similar doses, the results do suggest that the active ingredient in the extract of *C orbiculata* might not act similarly to pladienolide B on SF3B.

We found that apoptosis induction is replicated by isoform specific knockdown of hnRNPB1. RT-PCR revealed that knockdown of hnRNPB1 can increase Bcl-xs in colon cancer cells resulting in an increase in caspase-3 cleavage and a decrease in cell viability, suggesting that hnRNPB1 modulates splicing of BCL2L1 and is functionally involved in cell proliferation in HCT116 colon cancer cells. Although the splicing change of BCL2L1 is small between hnRNPB1 siRNA transfected HCT116 colon cancer cells when compared to the untransfected cell, the ratio of Bcl-xs/Bcl-xl could be critical for cell survival as evident by increased caspase-3-cleavage and decreased cell viability in hnRNPB1 siRNA transfected cells. A delicate balance between the two isoforms could determine the fate of cells, which may explain why the regulation of BCL2L1 alternative splicing is complex (57, 58). To confirm this, we showed that over-expression of hnRNPB1 blocks the effect of *C. orbiculata* extract in HCT116 colon cancer cells, indicating that the effect was hnRNP splicing dependent, and that hnRNPA2/B1 splicing is the target for the active ingredient(s) in *C. orbiculata* extract. The binding of hnRNPA2B1 with BCL2L1 and additional mRNAs (BCL2, MDM2, cMYC, CD44, CDK6, cJun, and TXNP) was further confirmed by RNA immunoprecipitation (RIP) assay. Given there are 10,459 alternative splicing events and hnRNPA2B1 interacts with multiple apoptotic and cancer genes it is probable that modulation of many of those genes collectively contribute to the reduced viability by *C. orbiculata* extract. Thus, while these results indicate that *C. orbiculata* extract enhances the binding of hnRNPA2B1 to Bclx pre-mRNA, they also suggest that further mechanisms through which control of hnRNPA2B1 splicing could regulate apoptosis, for instance by regulating BCL2, and further work will be required to quantitatively understand the contribution of Bclx splicing vs. other mechanisms.

Our interpretations depend upon a number of assumptions inherent in the methodology we used. These include the use of agarose gel electrophoresis and densitometry for the quantification of the relative abundance of the specific isoforms to confirm the change in splicing induced by *C. orbiculata* seen by RNASeq. There are multiple ways to determine RNA expression, including qRT-PCR, digital droplet PCR, northern blotting, and RNAse protection assay. Each of these has their advantages and disadvantages, particularly for examining alternative splicing. We have used conventional RT-PCR followed by separation by agarose gel electrophoresis so that both isoforms can be amplified in the same reaction, removing inter-sample variation, and comparing the intensity of each band to the other within a single reaction. While this removes the variation of differential logarithmic amplification between primers and reactions seen with qRT-PCR (59) and the potential for mispriming seen in qRT-PCR (60), it also introduces the potential for saturation of the PCR reaction, restriction of the dynamic range of the detectors, and potential for bleed through of signal from closely related bands. Further work on the level of isoforms to further confirm the sequencing results would be beneficial, potentially once the active ingredient in *C. orbiculata* has been identified, using digital droplet PCR. The results also assume that the change in number of transcripts is a consequence of alternative splicing, rather than differential degradation or enhanced stability of one isoform over another. This could be further investigated using RNAse protection assays as previously shown for other isoform changes (e.g., TIA-1) (61).

## Conclusion

Our findings on splicing of hnRNPA2B1 and BCL2L1 in HCT116 colon cancer cells and OE33 and KYSE70 esophageal cancer cells treated with the crude extract of *C. orbiculata* clearly suggests that *C. orbiculata* switches splicing of hnRNPA2B1, which in turn regulates splicing of BCLX to induce apoptosis in HCT116 colon cancer cells. It is clear that hnRNPB1 is critical for cell viability and apoptosis induction. These findings raise the interesting possibility that targeting hnRNPA2B1 splicing in colon cancer can be a useful therapeutic strategy to induce apoptosis and restrain proliferation and tumor progression. This study provides original insights into the possible use of *C. orbiculata*, a South African medicinal plant in the treatment of colon cancer. Additionally, taken together, our experimental evidence suggests a possible role of the hnRNPB1 in the tumorigenic potential of colon cancer. Therefore, there is a possibility of modulating hnRNPA2B1-regulated genes by manipulating the level or activity of hnRNPB1. The next step will be to evaluate how splicing changes impacts these genes and to isolate and characterize additional phytocompounds form the crude extract of *C. orbiculata*.

## Data Availability Statement

The datasets presented in this study can be found in online repositories. The names of the repository/repositories and accession number(s) can be found here: the NCBI Gene Expression Omnibus (GSE156221).

## Author Contributions

DB and ZD: concept and design. TM, MMb, KY-U, DH, AH, ZB, MMe, and NM: methodology and data acquisition. DH, TM, MMe, KY-U, AH, DH, ZB, NM, and ZD: analysis and interpretation of data (e.g., image analysis, statistical analysis etc.). TM, MMb, KY-U, DH, ZB, MMe, NM, ZD, and DB: writing, review and revision of the manuscript. DB and ZD: study supervision. All authors contributed to the article and approved the submitted version.

## Conflict of Interest

The authors declare that the research was conducted in the absence of any commercial or financial relationships that could be construed as a potential conflict of interest.

## References

[1] Cunningham D Atkin W Lenz HJ Lynch HT Minsky B Nordlinger B. Colorectal cancer. Lancet. (2010) 375:1030–47. 10.1016/S0140-6736(10)60353-4 20304247

[2] Bray F Ferlay J Soerjomataram I Siegel RL Torre LA Jemal A. Global cancer statistics 2018: GLOBOCAN estimates of incidence and mortality worldwide for 36 cancers in 185 countries. CA Cancer J Clin. (2018) 68:394–424. 10.3322/caac.21492 30207593

[3] Stintzing S. Management of colorectal cancer. F1000 Prime Rep. (2014) 6:108. 10.12703/P6-108 25580262 PMC4229728

[4] Brand M Gaylard P Ramos J. Colorectal cancer in South Africa: an assessment of disease presentation, treatment pathways and 5-year survival. S Afr Med J. (2018) 108:118–22. 10.7196/SAMJ.2017.v108i2.12338 29429443

[5] Loots E Sartorius B Madiba T Mulder C Clarke D. Is clinical research in oesophageal cancer in South Africa in crisis? A systematic review. World J Surg. (2017) 41:810–6. 10.1007/s00268-016-3778-5 27807706

[6] Lehnert M. Clinical multidrug resistance in cancer: a multifactorial problem. Eur J Cancer. (1996) 32:912–20. 10.1016/0959-8049(96)00069-X 8763332

[7] Mattern J. Drug resistance in cancer: a multifactorial problem. Anticancer Res. (2003) 23:1769–72. 12820456

[8] Schwerk C Schulze-Osthoff K. Regulation of apoptosis by alternative pre-mRNA splicing. Mol Cell. (2005) 19:1–13. 10.1016/j.molcel.2005.05.026 15989960

[9] Mercatante DR Bortner CD Cidlowski JA Kole R. Modification of alternative splicing of Bcl-x pre-mRNA in prostate and breast cancer cells analysis of apoptosis and cell death. J Biol Chem. (2001) 276:16411–7. 10.1074/jbc.M009256200 11278482

[10] Kim E Goren A Ast G. Insights into the connection between cancer and alternative splicing. Trends Genet. (2008) 24:7–10. 10.1016/j.tig.2007.10.001 18054115

[11] Marzese DM Manughian-Peter AO Orozco JI Hoon DS. Alternative splicing and cancer metastasis: prognostic and therapeutic applications. Clin Exp Metastasis. (2018) 35:393–402. 10.1007/s10585-018-9905-y 29845349

[12] Anczuków O Krainer AR. Splicing-factor alterations in cancers. RNA. (2016) 22:1285–301. 10.1261/rna.057919.116 27530828 PMC4986885

[13] Zong Z Li H Yi C Ying H Zhu Z Wang H. Genome-wide profiling of prognostic alternative splicing signature in colorectal cancer. Front Oncol. (2018) 8:537. 10.3389/fonc.2018.00537 30524964 PMC6262947

[14] Mizui Y Sakai T Iwata M Uenaka T Okamoto K Shimizu H Pladienolides, new substances from culture of streptomyces platensis Mer-11107. J Antibiot. (2004) 57:188–96. 10.7164/antibiotics.57.188 15152804

[15] Kotake Y Sagane K Owa T Mimori-Kiyosue Y Shimizu H Uesugi M. Splicing factor SF3b as a target of the antitumor natural product pladienolide. Nat Chem Biol. (2007) 3:570. 10.1038/nchembio.2007.16 17643112

[16] Yoon S-O Shin S Lee H-J Chun H-K Chung A-S. Isoginkgetin inhibits tumor cell invasion by regulating phosphatidylinositol 3-kinase/Akt–dependent matrix metalloproteinase-9 expression. Mol Cancer Ther. (2006) 5:2666–75. 10.1158/1535-7163.MCT-06-0321 17121913

[17] Russo M Spagnuolo C Tedesco I Russo GL Phytochemicals in cancer prevention and therapy: truth or dare? Toxins. (2010) 2:517–51. 10.3390/toxins2040517 22069598 PMC3153217

[18] Benarba B Pandiella A. Colorectal cancer and medicinal plants: principle findings from recent studies. Biomed Pharmacother. (2018) 107:408–23. 10.1016/j.biopha.2018.08.006 30099345

[19] Noble R Beer C Cutts J. Role of chance observations in chemotherapy: Vinca rosea. Ann NY Acad Sci. (1958) 76:882–94. 10.1111/j.1749-6632.1958.tb54906.x 13627916

[20] Jordan MA Wilson L. Microtubules as a target for anticancer drugs. Nat Rev Cancer. (2004) 4:253. 10.1038/nrc1317 15057285

[21] Minocha A Long BH. Inhibition of the DNA catenation activity of type II topoisomerase by VP16-213 and VM26. Biochem Biophys Res Commun. (1984) 122:165–70. 10.1016/0006-291X(84)90454-6 6331440

[22] Imbert T. Discovery of podophyllotoxins. Biochimie. (1998) 80:207–22. 10.1016/S0300-9084(98)80004-7 9615861

[23] Wall ME Wani M Cook C Palmer KH McPhail AT Sim G Plant antitumor agents. I. the isolation and structure of camptothecin, a novel alkaloidal leukemia and tumor inhibitor from camptotheca acuminata1, 2. J Am Chem Soc. (1966) 88:3888–90. 10.1021/ja00968a057

[24] Bissery M-C Guénard D Guéritte-Voegelein F Lavelle F. Experimental antitumor activity of taxotere (RP 56976, NSC 628503), a taxol analogue. Cancer Res. (1991) 51:4845–52. 1680023

[25] Mita AC Denis LJ Rowinsky EK DeBono JS Goetz AD Ochoa L. Phase I and pharmacokinetic study of XRP6258 (RPR 116258A), a novel taxane, administered as a 1-hour infusion every 3 weeks in patients with advanced solid tumors. Clin Cancer Res. (2009) 15:723–30. 10.1158/1078-0432.CCR-08-0596 19147780

[26] Ojima I Lichtenthal B Lee S Wang C Wang X Taxane anticancer agents: a patent perspective. Expert Opin Ther Pat. (2016) 26:1–20. 10.1517/13543776.2016.1111872 26651178 PMC4941984

[27] Falzone L Salomone S Libra M. Evolution of cancer pharmacological treatments at the turn of the third millennium. Front Pharmacol. (2018) 9:1300. 10.3389/fphar.2018.01300 30483135 PMC6243123

[28] Dobin A Davis CA Schlesinger F Drenkow J Zaleski C Jha S. STAR: ultrafast universal RNA-seq aligner. Bioinformatics. (2013) 29:15–21. 10.1093/bioinformatics/bts635 23104886 PMC3530905

[29] Liao Y Smyth GK Shi W. featureCounts: an efficient general purpose program for assigning sequence reads to genomic features. Bioinformatics. (2014) 30:923–30. 10.1093/bioinformatics/btt656 24227677

[30] Robinson MD McCarthy DJ Smyth GK. edgeR: a bioconductor package for differential expression analysis of digital gene expression data. Bioinformatics. (2010) 26:139–40. 10.1093/bioinformatics/btp616 19910308 PMC2796818

[31] Lohse M Bolger AM Nagel A Fernie AR Lunn JE Stitt M. RobiNA: a user-friendly, integrated software solution for RNA-Seq-based transcriptomics. Nucl Acids Res. (2012) 40:W622–7. 10.1093/nar/gks540 22684630 PMC3394330

[32] Shen S Park JW Lu ZX Lin L Henry MD Wu YN. rMATS: robust and flexible detection of differential alternative splicing from replicate RNA-Seq data. Proc Natl Acad Sci USA. (2014) 111:E5593–601. 10.1073/pnas.1419161111 25480548 PMC4280593

[33] Liao Y Wang J Jaehnig EJ Shi Z Zhang B. WebGestalt 2019: gene set analysis toolkit with revamped UIs and APIs. Nucl Acids Res. (2019) 47:W199–205. 10.1093/nar/gkz401 31114916 PMC6602449

[34] Subramanian A Tamayo P Mootha VK Mukherjee S Ebert BL Gillette MA. Gene set enrichment analysis: a knowledge-based approach for interpreting genome-wide expression profiles. Proc Natl Acad Sci USA. (2005) 102:15545–50. 10.1073/pnas.0506580102 16199517 PMC1239896

[35] Dai S Zhang J Huang S Lou B Fang B Ye T. HNRNPA2B1 regulates the epithelial–mesenchymal transition in pancreatic cancer cells through the ERK/snail signalling pathway. Cancer Cell Int. (2017) 17:12. 10.1186/s12935-016-0368-4 28077929 PMC5223355

[36] Boise LH González-García M Postema CE Ding L Lindsten T Turka LA. bcl-x, a bcl-2-related gene that functions as a dominant regulator of apoptotic cell death. Cell. (1993) 74:597–608. 10.1016/0092-8674(93)90508-N 8358789

[37] Lebedeva I Rando R Ojwang J Cossum P Stein C. Bcl-xL in prostate cancer cells: effects of overexpression and down-regulation on chemosensitivity. Cancer Res. (2000) 60:6052–60. 11085527

[38] Ladomery M. Aberrant alternative splicing is another hallmark of cancer. Int J Cell Biol. (2013) 2013:463786. 10.1155/2013/463786 24101931 PMC3786539

[39] Plötz M Gillissen B Hossini A Daniel P Eberle J. Disruption of the VDAC2–Bak interaction by Bcl-x S mediates efficient induction of apoptosis in melanoma cells. Cell Death Differ. (2012) 19:1928. 10.1038/cdd.2012.71 22705850 PMC3504705

[40] Warren CF Wong-Brown MW Bowden NA. BCL-2 family isoforms in apoptosis and cancer. Cell Death Dis. (2019) 10:177. 10.1038/s41419-019-1407-6 30792387 PMC6384907

[41] Badrichani A Stroka D Bilbao G Curiel D Bach F Ferran C. Bcl-2 and Bcl-x L serve an anti-inflammatory function in endothelial cells through inhibition of NF-κB. J Clin Invest. (1999) 103:543–53. 10.1172/JCI2517 10021463 PMC408093

[42] Choi S Chen Z Tang LH Fang Y Shin SJ Panarelli NC. Bcl-xL promotes metastasis independent of its anti-apoptotic activity. Nat Commun. (2016) 7:10384. 10.1038/ncomms10384 26785948 PMC4735924

[43] de Carné Trécesson S Souazé F Basseville A Bernard A-C Pécot J Lopez J. BCL-X L directly modulates RAS signalling to favour cancer cell stemness. Nat Commun. (2017) 8:1123. 10.1038/s41467-017-01079-1 29066722 PMC5654832

[44] Hu Y Sun Z Deng J Hu B Yan W Wei H. Splicing factor hnRNPA2B1 contributes to tumorigenic potential of breast cancer cells through STAT3 and ERK1/2 signaling pathway. Tumor Biol. (2017) 39:1010428317694318. 10.1177/1010428317694318 28351333

[45] Han SP Tang YH Smith R. Functional diversity of the hnRNPs: past, present and perspectives. Biochem J. (2010) 430:379–92. 10.1042/BJ20100396 20795951

[46] Golan-Gerstl R Cohen M Shilo A Suh S-S Bakàcs A Coppola L. Splicing factor hnRNP A2/B1 regulates tumor suppressor gene splicing and is an oncogenic driver in glioblastoma. Cancer Res. (2011) 71:4464–72. 10.1158/0008-5472.CAN-10-4410 21586613

[47] Kozu T Henrich B Schäfer KP. Structure and expression of the gene (HNRPA2B1) encoding the human hnRNP protein A2/B1. Genomics. (1995) 25:365–71. 10.1016/0888-7543(95)80035-K 7789969

[48] Sueoka E Goto Y Sueoka N Kai Y Kozu T Fujiki H. Heterogeneous nuclear ribonucleoprotein B1 as a new marker of early detection for human lung cancers. Cancer Res. (1999) 59:1404–7. 10197602

[49] Pu D Wang Y Li W Wen C Zhou T Chen M The expression inhibition of HnRNP B1 in lung cell line A549 by small interfering RNA. Lab Med. (2009) 40:283–9. 10.1309/LMX7EGYSW9ZDC5WG

[50] Wu S Sato M Endo C Sakurada A Dong B Aikawa H. hnRNP B1 protein may be a possible prognostic factor in squamous cell carcinoma of the lung. Lung Cancer. (2003) 41:179–86. 10.1016/S0169-5002(03)00226-5 12871781

[51] Aird D Teng T Huang C-L Pazolli E Banka D Cheung-Ong K. Sensitivity to splicing modulation of BCL2 family genes defines cancer therapeutic strategies for splicing modulators. Nat Commun. (2019) 10:137. 10.1038/s41467-018-08150-5 30635584 PMC6329755

[52] Van Wyk B-E Oudtshoorn Bv Gericke N Medicinal Plants of South Africa. Pretoria: Briza Publications (1997).

[53] Amabeoku G Green I Kabatende J. Anticonvulsant activity of *Cotyledon orbiculata* L. (*Crassulaceae*) leaf extract in mice. J Ethnopharmacol. (2007) 112:101–7. 10.1016/j.jep.2007.02.016 17398051

[54] van Wyk B-E Gericke N People's Plants: A Guide to Useful Plants of Southern Africa. Pretoria: Briza Publications (2000).

[55] Wang J Lenardo MJ. Roles of caspases in apoptosis, development, and cytokine maturation revealed by homozygous gene deficiencies. J Cell Sci. (2000) 113:753–7. 10671365 10.1242/jcs.113.5.753

[56] Zheng TS Schlosser SF Dao T Hingorani R Crispe IN Boyer JL. Caspase-3 controls both cytoplasmic and nuclear events associated with Fas-mediated apoptosis *in vivo*. Proc Natl Acad Sci USA. (1998) 95:13618–23. 10.1073/pnas.95.23.13618 9811849 PMC24868

[57] Paronetto MP Achsel T Massiello A Chalfant CE Sette C. The RNA-binding protein Sam68 modulates the alternative splicing of Bcl-x. J Cell Biol. (2007) 176:929–39. 10.1083/jcb.200701005 17371836 PMC2064079

[58] Moore MJ Wang Q Kennedy CJ Silver PA. An alternative splicing network links cell-cycle control to apoptosis. Cell. (2010) 142:625–36. 10.1016/j.cell.2010.07.019 20705336 PMC2924962

[59] Camacho Londono J Philipp SE. A reliable method for quantification of splice variants using RT-qPCR. BMC Mol Biol. (2016) 17:8. 10.1186/s12867-016-0060-1 26979160 PMC4793508

[60] Bates DO Mavrou A Qiu Y Carter JG Hamdollah-Zadeh M Barratt S. Detection of VEGF-A(xxx)b isoforms in human tissues. PLoS ONE. (2013) 8:e68399. 10.1371/journal.pone.0068399 23935865 PMC3729684

[61] Hamdollah Zadeh MA Amin EM Hoareau-Aveilla C Domingo E Symonds KE Ye X. Alternative splicing of TIA-1 in human colon cancer regulates VEGF isoform expression, angiogenesis, tumour growth and bevacizumab resistance. Mol Oncol. (2015) 9:167–78. 10.1016/j.molonc.2014.07.017 25224594 PMC4286123

