# Supplementary figures and images for: Apoptosis in Cancer Cells Is Induced by Alternative Splicing of hnRNPA2/B1 Through Splicing of Bcl-x, a Mechanism that Can Be Stimulated by an Extract of the South African Medicinal Plant, Cotyledon orbiculata
Source: Front Oncol. 2020 Oct 8;10:547392. doi: 10.3389/fonc.2020.547392 (PMC7580256; doi:10.3389/fonc.2020.547392)

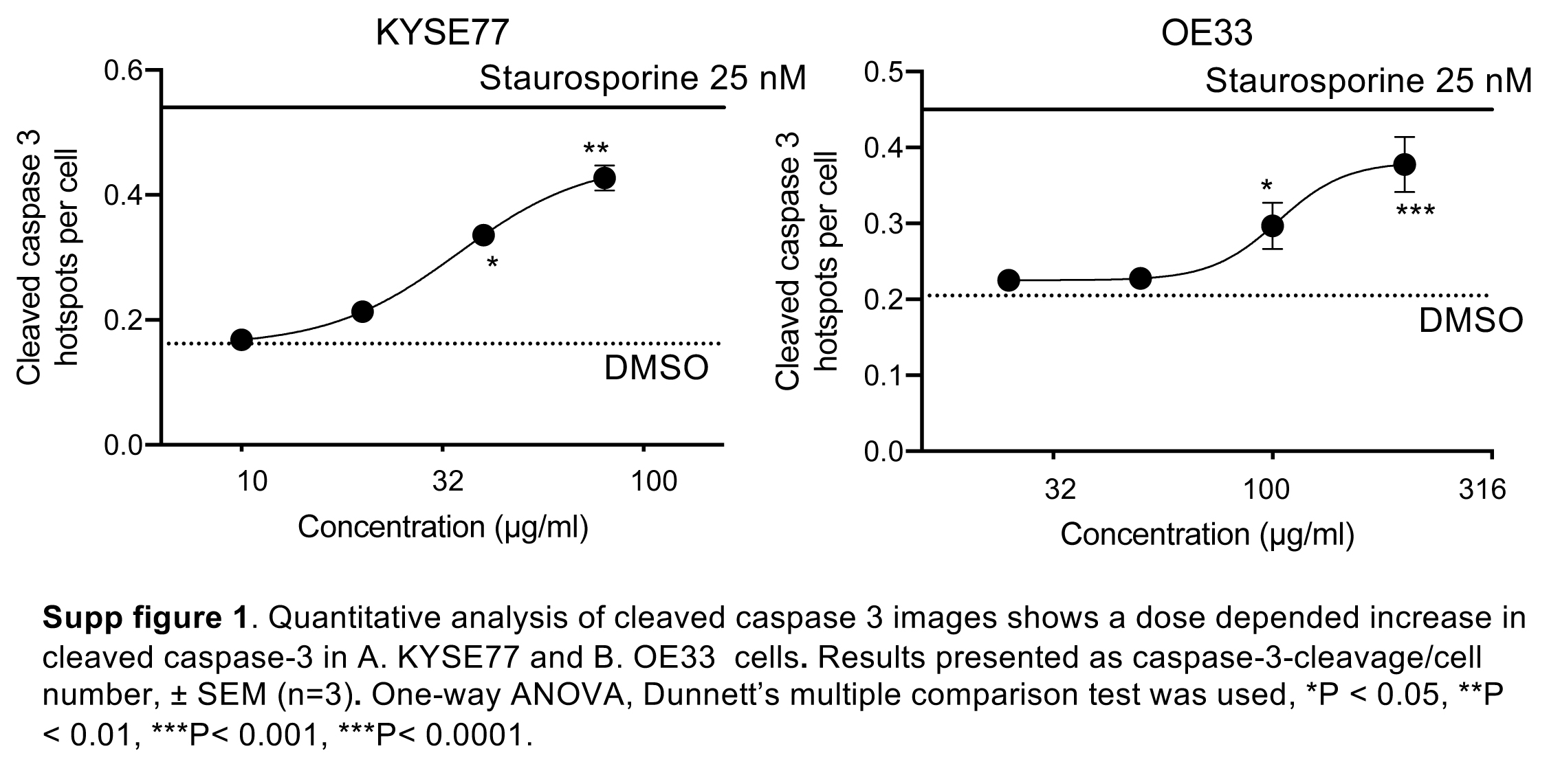

Supplement: Supplementary file 1 [file Image_1.JPEG]
